# A patient diagnosed with new-onset type 1 diabetes and Addison’s disease at initial presentation

**DOI:** 10.1530/EDM-23-0106

**Published:** 2024-05-13

**Authors:** Emma Towslee, Adrienne Macdonald, Zohreh Shoar

**Affiliations:** 1 Cottage Children’s Medical Center, Santa Barbara, California, USA

**Keywords:** Adolescent/young adult, Female, White, United States, Adrenal, Diabetes, Addison's disease, Unique/unexpected symptoms or presentations of a disease, May, 2024

## Abstract

**Summary:**

A previously healthy 17-year-old female presented to the emergency department with complaints of vomiting, shortness of breath, and tachycardia. She was found to have an elevated blood glucose and was admitted for presumed new onset type 1 diabetes mellitus (T1DM). During the admission, she was noted to have frequent episodes of hypoglycemia despite conservative insulin dosing and high urine output with glucosuria, which seemed out of proportion to her glucose levels and fluid status. She also had persistent hyponatremia despite normalization of blood glucose. Further work-up was initiated to investigate alternative or additional diagnoses to explain these atypical findings. Adrenocorticotropic hormone (ACTH) level was elevated, consistent with the diagnosis of Addison’s disease, which led to the subsequent diagnosis of autoimmune polyglandular syndrome type II (APS-2). This is one of the first reports in the literature of concurrent diagnosis of T1DM and Addison’s disease at initial presentation and demonstrates the importance of not anchoring to one diagnosis.

**Learning points:**

- This case shows the importance of considering multiple diagnoses and investigating atypical signs and symptoms.
- This case highlights the importance of a thorough history including review of systems.
- Hyponatremia and recurrent hypoglycemia in a person with type 1 diabetes should raise suspicion for adrenal insufficiency.
- This case makes us consider the screening for Addison’s disease in a person with new onset type 1 diabetes in addition to autoimmune thyroid disease and celiac disease.
- People with an autoimmune disease should be monitored for other autoimmune diseases in the future.

## Background

The prevalence of type 1 diabetes mellitus (T1DM) is relatively common in the pediatric population and is about 2.6 per 1000 in the entire civilian noninstitutionalized US population (1), while the prevalence of Addison’s disease is between 40 and 60 people per million of the general population (2). Autoimmune polyglandular syndrome type 2 (APS-2) is a rare clustering of autoimmune disorders with a reported prevalence ranging from 1:1000 to 1:20 000 in the general population (3). Our case is novel because it is the second reported case of concurrent diagnosis of T1DM and Addison’s disease diagnosed in the same hospitalization and the concurrent diagnosis of APS 2. Additionally, it is interesting to note that our patient was diagnosed early, preventing life-threatening Addisonian crisis.

## Case presentation

A previously healthy 17-year-old female presented to urgent care with complaints of 1 day of vomiting and intermittent shortness of breath with associated chest tightness over the past 3 months. Her EKG was found to be normal. A comprehensive metabolic panel revealed an elevated blood glucose of 25.2 mmol/L (453 mg/dL) and HbA1C was elevated at 121 g/L (12%). Review of symptoms revealed additional symptoms including fatigue, polyuria, polydipsia, and weight loss.

She was admitted to the pediatric service for presumed new onset T1DM. There was no family history of diabetes. Her maternal grandmother did have hypothyroidism. Of note, the patient did have a history of Influenza A about 6 months prior to her presentation. On further review of her past medical history, she was noted to have growth delay starting about 2 years prior to presentation. She was below the first percentile for weight and at the first percentile for height. Her mid-parental height estimated she should have been at the tenth percentile. Mother also reported that the patient seemed to tan easily. The patient denied amenorrhea or irregular menstrual cycles.

Over the past year, her school performance declined from straight ‘A’s’ to failing. In addition, her maternal grandfather had recently passed away and her single mother was the sole caregiver for the patient’s maternal grandmother.

On admission her BMI was at the fifth percentile at 17.3 kg/m^2^. Her initial blood pressure was 95/60 (normal: 90–120/ 55–80), and during the admission, it remained within normal limits. Her other vital signs were otherwise normal including a heart rate of 80 bpm, normal respiratory rate, and 100% SpO2 on room air. On physical examination, she was thin and pale without evidence of hyperpigmentation. Her mucous membranes were dry, her abdomen was soft, and cardiac examination revealed a regular rate and rhythm. Her lungs were clear to auscultation. She had a normal neurologic exam and was normal mentation.

**Figure 1:**
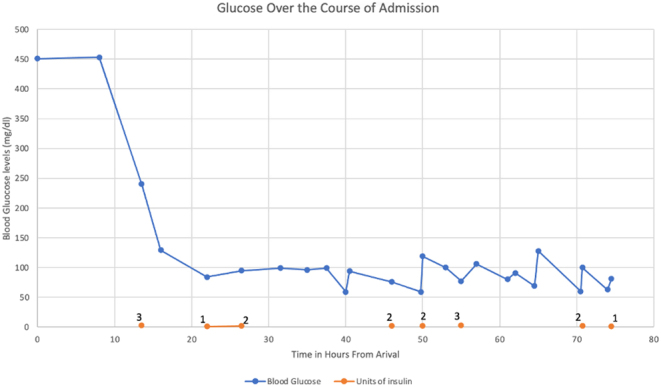
Patient’s glucose levels during her admission. Lispro (meals) and nightly Lantus doses are indicated at the bottom of the graph.

She was not in diabetic ketoacidosis (DKA) as her laboratory evaluation showed a normal anion gap at 9 mmol/L and bicarbonate was normal of 24 mEq/L (Table 1). Her C-peptide was low at 0.14 nmol/L (0.42 ng/mL) and her diabetes antibodies later revealed a positive for zinc transporter 8 (ZNT8) antibodies and islet antigen 2 (IA-2) antibodies (see Fig. 1 for values).

**Table 1:** Pertinent lab values during admission and after patient was discharged.

Labs	Normal range	Day 1	Day 2	Day 3	8 weeks	15 weeks	17 weeks
Blood glucose							
mg/dL	70–100	453	145				
mmol/L	3.9– 5.6	25.2	8.05				
Hemoglobin A1C							
%	<5.7	12.1			8.2		
g/dL	<57	121			82		
C-peptide							
ng/mL	0.81–3.85	0.42					
nmol/L	0.27–1.27	0.14					
Sodium (mmol/L)	134–145	126	133				
Potassium (mmol/L)	3.5–5.1	4.4	4				
Beta-hydroxybutyrate							
mg/dL	<3.1	22.3					
mmol/L	<0.1	0.73					
Anion gap (mmol/L)	3–14	9	6				
Creatinine							
mg/dL	0.5–1.1	0.83		0.56			
μmol/L	44–97	73.4		49.5			
WBC count (10^3^/μL or 10^9^/L)	4.0–10.0	9.0					
RBC count (10^6^/μL or 10^12^/L)	3.77–5.19	5.07					
Hemoglobin							
g/dL	11–15.5	14.4					
mmol/L	6.8–9.6	8.9					
Hematocrit (%)	34.9–47	39.70					
MCV (fL)	81.3–101.6	78.3					
Platelet count (μL)	150 000–450 000	371 000					
TSH (μlU/mL or mIU/L)	0.480–4.170	7.643					2.716
Thyroxine							
ng/mL	0.83–1.43	1.33					
pmol/L	10.7–18.4	17.1					
ACTH							
pg/mL	7.2–63.3			1,500		11.2	
pmol/L	1.59–13.94			330		2.47	
Renin (ng/mL/h or μg/h/L)	0.167–5.380			14.727		0.276	
TPA(IU/mL or kIU/L)	<60	42					
TA (IU/mL or kIU/L)	<60	<15					
Tissue TGA (IU/mL or kIU/L)							
IgA	0–3	<2					
IgG	0–3	<2					
Anti-GAD 65 Ab (U/mL or kU/L)	<5	<5					
Insulin Ab (U/mL or kU/L)	<5	<					
IA-2 AutoAb (U/mL or kU/L)	<7.5	40					
ZNT8 Ab (U/mL or kU/L)	<15	>500					
21- Hydroxylase Ab	Negative	Positive					

Ab, antibodies; MCV, mean corpuscular volume; RBC, red blood cell; TA, thyroglobulin antibody; TGA, transglutaminase antibody; TPA, thyroid peroxidase antibody; WBC, white blood cell.

## Investigation

**Figure 2:**
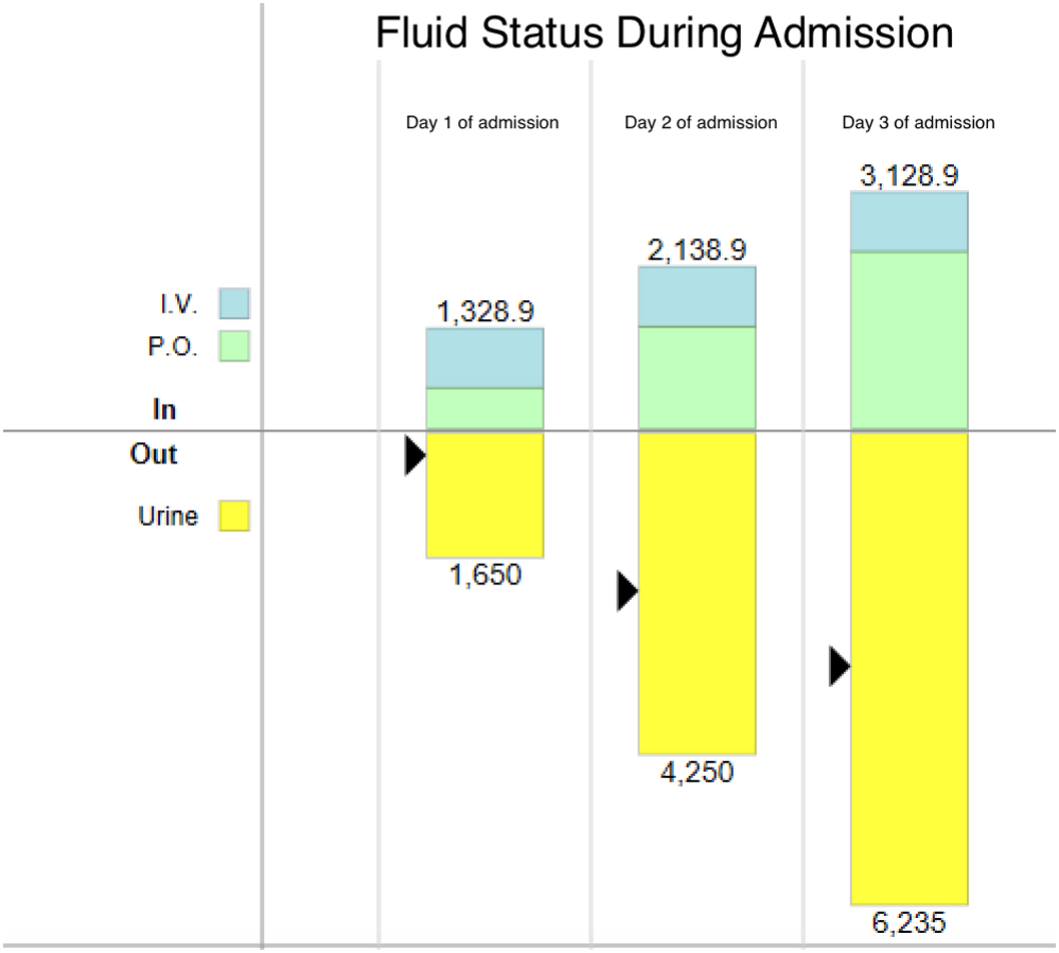
Patient’s urinary output. The patient’s total input and output values are for each day of admission and reported in milliliters. On the third day of admission, the patient’s daily urine output was 3.5 mL/kg/h (normal: 0.5–1.5 mL/kg/h). During the admission, her fluid balance was negative 2.4 L.

Throughout her stay, she was noted to have frequent episodes of hypoglycemia, particularly in the early morning hours, despite a conservative insulin dose (Fig. 1). In addition, she had high urine output of 5.7 mL/kg/h (normal: 0.5–1.5 mL/kg/h) with glucosuria despite euglycemia and minimal intravenous fluids (Fig. 2). She also had continued mild hyponatremia despite normalization of blood sugar; her initial sodium level was 126 mmol/L that was corrected to 132 mmol/L. After achieving euglycemia with a blood sugar of 8.05 mmol/L (145 mg/dL), her sodium remained low at 133 mmol/L (133 mEq/L) with a potassium of 4 mmol/L (4 mEq/L). In addition, her blood pressure continued to be on the lower end of normal with a systolic blood pressure ranging between 89 and 103 and a diastolic between 56 and 68.

Given her atypical course and persistent findings, additional diagnoses including Fanconi syndrome, early honeymoon period, psychogenic polydipsia, and Addison’s disease were considered. Fanconi syndrome is a disorder that impacts the kidneys’ ability to resorb specific essential substances, leading to their elimination from the body through urine. Common symptoms of this condition include increased urination, excessive thirst, short stature, and musculoskeletal pain. A urinary phosphate was normal, ruling out Fanconi syndrome. Early honeymoon period typically occurs after weeks up to 3 months of treatment, so that seemed unlikely to be the case (4). She was not observed to have high fluid intake and her mother denied a history of drinking excessively, so this was unlikely to be psychogenic polydipsia. An ACTH level was obtained initially mid-day and later repeated in the early morning was over 330 pmol/L (1500 pg/mL), concerning for Addison’s disease. Her initial renin level came back elevated at 14.727 µg/h/L (14.727 ng/mL/h) and she was positive for adrenal 21-hydroxylase antibodies, providing further evidence for Addison’s disease.

## Treatment

Our patient was treated with both short-acting and long-acting insulin for her diabetes. Specifically, she was discharged on Lantus (glargine) 8 units nightly and Humalog (lispro) with an insulin to carbohydrate ratio of 1:20 and a correction factor of 1:50. To treat her Addison’s disease she was also started on hydrocortisone 10mg (15.6 mg/BSA) once in the morning and once at night, and fludrocortisone 0.1 mg once daily. The standard maintenance steroid dosing for Addison’s disease in children is 10–12 mg/m^2^/day. Because her ACTH levels were so high, she was started on a higher dose. This dose has been managing her symptoms well thus far. If she was to develop an acute illness, was advised to double her daily dose. She was also educated and provided a prescription for injectable hydrocortisone for severe stress, decompensation, and pre-surgery.

### Outcome and follow-up

In the 2 months following her diagnosis, she has gained 3 kg in 2 months and is now at the first percentile for weight. Her blood sugars have improved and her HbA1C is now 82 g/L (8.2%). Her blood pressure has also improved and normalized with systolic blood pressure between 117 and 118 and diastolic blood pressure between 68 and 81. Her ACTH and renin have normalized to 2.47 pmol/L (11.2 pg/mL) and 0.276 µg/h/L (0.276 ng/mL/h) respectively. Symptomatically, she has improved; however, given the diagnosis two autoimmune disorders requiring lifelong treatment, she has benefited from the emotional support from a counselor to cope with her new diagnoses and psychosocial stressors at home.

## Discussion

T1DM is a relatively common diagnosis in pediatrics, with the incidence increasing 3-4% over the past 30 years (5). A standard work-up for new onset T1DM includes screening for celiac disease and autoimmune thyroid disease, due to the known increased risk for developing multiple autoimmune disorders. Concurrent diagnosis of Addison’s disease along with either T1DM or autoimmune thyroid disease is consistent with APS-2 (6). APS-2 is a much rarer disorder occurring in 40–60 per million people. APS-2 is a polygenic autoimmune disorder, marked by considerable diversity attributed to multiple genetic loci and environmental factors, leading to organ-specific damage through lymphocytic infiltration. Individuals with APS-2 are usually diagnosed between 20 and 40 years of age and women are three times more likely to be affected. The diagnosis of APS-2 is often delayed due to the diverse and varied presentation in many cases (3).

Our patient’s thyroid-stimulating hormone (TSH) was mildly elevated at 7.643 mlU/L and thyroxine was normal at 17.1 pmol/L (1.33 ng/mL). However, given her TSH normalized weeks later and she had neither thyroglobulin antibodies nor thyroid peroxidase antibodies, this was thought to represent euthyroid sick syndrome rather than hypothyroidism.

The pathophysiology of Addison’s disease is the result of circulating 21-hydroxylase antibodies which inhibit the 21-hydroxylase enzyme, triggering a downward cascade which ultimately leads to the lack of production of cortisol and aldosterone within the adrenal gland and a compensatory increase in ACTH. This downward cascade is controlled by the hypothalamic–pituitary–adrenal axis. The hypothalamus will produce corticotropin-releasing hormone (CRH) and then signals the pituitary gland to produce ACTH. ACTH then signals the adrenal glands to produce cortisol. Without cortisol to negatively provide feedback on the pituitary gland, ACTH continues to rise. Given our patient had elevated ACTH along with positive adrenal and 21-hydralase antibodies, our patient likely has Addison’s disease.

Cortisol deficiency can lead to symptoms of fatigue, hypotension, and hypoglycemia, while lack of aldosterone leads to hyponatremia with resulting polyuria. The hyperpigmentation in Addison’s disease is due to elevated production of α-melanocyte-stimulating hormone (αMSH). Pro-hormone peptide pro-opiomelanocortin (POMC) is the source of both αMSH and ACTH. In primary adrenal insufficiency, there is a significant increase in POMC production as a response to decreased cortisol levels, leading to the release of αMSH and causing the subsequent hyperpigmentation (7). Though our patient did not show signs of hyperpigmentation on initial exam, her mother did report a history of ‘easy tanning’.

Addison’s disease typically presents in life-threatening adrenal crisis. Patients with T1DM have a 10% increased risk of developing Addison’s disease in their lifetime (8). This case report highlights that a high index of suspicion for another autoimmune diseases is important in managing patients with T1DM such as thyroid disease and celiac disease. Because of the increased risk in patients with TIDM and the risk of presenting in adrenal crisis, it may be warranted to screen for concurrent Addison’s disease in those with unexplained persistent electrolyte abnormalities.

The current Endocrine Society guidelines recommend screening patients who may have Addison’s disease with both an early morning ACTH level and cortisol level to evaluate for glucocorticoid deficiency. It is also recommended to obtain an aldosterone and renin level to evaluate for mineralocorticoid deficiency (9). However, those with very subtle findings such as mild hyponatremia, polyuria despite euglycemia, and frequent hypoglycemia, like our patient, may benefit from early screening with 21 hydroxylase antibodies. We hypothesize this test may catch low risk individuals and may be easier for patients to obtain compared to a morning cortisol test which should be done between 07:00 h and 09:00 h. A morning cortisol test has a 71.88% sensitivity when using values less than 5 µg/dL, while testing for 21 hydroxylase antibodies has an 87.0% sensitivity with an index value of <45 as a negative cutoff (10, 11). However, more studies need to be done to evaluate its cost effectiveness. The risk of death from adrenal crisis is about 6% (12). Early detection could help prevent adrenal crisis, reducing morbidity and mortality associated with Addison’s disease.

Additionally, screening for other autoimmune disorder, such as premature ovarian insufficiency (POI), should also be considered for all women with APS. There is an association of POI with APS, specifically in APS-1 and APS-2. About 10–25% of patients with APS-2 will develop POI (13). Therefore, monitoring patient’s menstrual cycles is critical and in those with APS-2 and irregular menstrual cycles, one could consider screening by obtaining an anti-Müllerian hormone level for risk assessment of developing POI (13). Additionally, other associated autoimmune diseases with APS-2 include pernicious anemia, celiac disease, alopecia, and vitiligo which should be considered and monitored.

This case is unique because both diseases were diagnosed at the same time of presentation. There are few cases that reported the concurrent diagnosis of T1DM and Addison’s disease at initial presentation. Our case report is novel compared to another reported case in the literature, in that our patient was not in diabetic ketoacidosis (DKA) and did not present with a severe presentation (14). The patient reported by Graf *et al.* was a male who had extreme hyponatremia with an initial sodium of 108 mmol//L that only improved to 119–122 mmol/L after hydration and blood glucose stabilization. That patient was in the ICU and did not have polyuria or hypoglycemia. Our female patient showed subtle signs including mild hyponatremia, polyuria, and hypoglycemia despite conservative insulin doses which lead to further work-up and investigation. It would be interesting to retrospectively evaluate if the other patient had similar subtle findings prior to his presentation. A limitation to this case report is that we did not obtain cortisol or aldosterone levels; however, based on our patient’s clinical presentation, elevated ACTH and renin levels, and 21-hydroxylase antibodies, she was diagnosed with Addison’s disease and responded well to treatment.

In conclusion, APS-2 is a rare autoimmune disorder that represents an uncommon diagnosis characterized by the coexistence of Addison’s disease with autoimmune thyroid disease, type 1 autoimmune diabetes mellitus, or a combination of both. Addison’s disease is also a rare diagnosis that is diagnosed by an elevated early morning ACTH level and low cortisol level or a cosyntropin stimulation test. However, in those who have subtle findings like our patient, one may consider ordering 21-hydroxylase antibodies as an initial test. More studies are needed to investigate the cost effectiveness and availability of the test to detect early onset Addison’s disease.

## Declaration of interest

The authors declare that there is no conflict of interest that could be perceived as prejudicing the impartiality of this case report.

## Funding

This study did not receive any specific grant from any funding in the public, commercial, or not-for-profit sector.

## Patient consent

Signed informed consent was obtained from the patient’s relatives or guardians.

## Author contribution statement

ET and AM were the physicians caring for the patient in the hospital and also wrote the manuscript.

## Acknowledgements

Thanks are due to Dr Jeffery Frazer, Dr Rachel Cohen, and Heather Stevens for their review of the manuscript.
